# Sex differences in functional outcomes of intravenous thrombolysis among patients with lacunar stroke

**DOI:** 10.3389/fneur.2024.1341423

**Published:** 2024-02-13

**Authors:** Patrizia Wueger, Roberta Noseda, Alberto Pagnamenta, Giovanni Bianco, David Seiffge, Patrik Michel, Krassen Nedeltchev, Leo Bonati, Georg Kägi, Julien Niederhauser, Thomas Nyffeler, Andreas Luft, Susanne Wegener, Ludwig Schelosky, Friedrich Medlin, Biljana Rodic, Nils Peters, Susanne Renaud, Marie Luise Mono, Emmanuel Carrera, Urs Fischer, Gian Marco De Marchis, Carlo W. Cereda

**Affiliations:** ^1^Faculty of Biomedical Sciences, Università Della Svizzera Italiana, Lugano, Switzerland; ^2^Division of Clinical Pharmacology and Toxicology, Institute of Pharmacological Sciences of Southern Switzerland, Ente Ospedaliero Cantonale, Lugano, Switzerland; ^3^Clinical Trial Unit, Ente Ospedaliero Cantonale, Lugano, Switzerland; ^4^Department of Intensive Care, Ente Ospedaliero Cantonale, Lugano, Switzerland; ^5^Division of Pneumology, University of Geneva, Geneva, Switzerland; ^6^Neurocentre of Southern Switzerland, Ospedale Regionale di Lugano, Ente Ospedaliero Cantonale, Lugano, Switzerland; ^7^Department of Neurology, University Hospital Bern, Bern, Switzerland; ^8^Stroke Center, Neurology Service, Lausanne University Hospital, Lausanne, Switzerland; ^9^Department of Neurology, Kantonsspital Aarau, Aarau, Switzerland; ^10^Department of Neurology and Stroke Center, University Hospital Basel and University of Basel, Basel, Switzerland; ^11^Department of Neurology, Kantonsspital St. Gallen, St. Gallen, Switzerland; ^12^Stroke Unit, Hopital Nyon, Nyon, Switzerland; ^13^Center of Neurology and Neurorehabilitation, Luzerner Kantonsspital, Luzern, Switzerland; ^14^Department of Neurology, Universitätsspital Zürich, Zürich, Switzerland; ^15^Division of Neurology, Kantonsspital Münsterlingen, Münsterlingen, Switzerland; ^16^Division of Neurology, HFR Fribourg, Stroke Unit, Fribourg, Switzerland; ^17^Department of Neurology, Kantonsspital Winterthur, Winterthur, Switzerland; ^18^Department of Neurology and Stroke Center, Hirslanden Hospital, Zurich, Switzerland; ^19^Division of Neurology, Pourtalès Hospital, Neuchatel, Switzerland; ^20^Stroke Unit, Stadtspital Waid und Triemli, Zürich, Switzerland; ^21^Department of Neurology, Hôpitaux Universitaires de Genève, Geneva, Switzerland

**Keywords:** lacunar stroke, intravenous thrombolysis, sex differences, functional outcome, cohort study

## Abstract

**Background:**

This study aimed to assess if there are sex differences in the functional outcome of intravenous thrombolysis (IVT) among patients with lacunar stroke (LS).

**Methods:**

Consecutive patients admitted from 1 January 2014 to 31 January 2020 to hospitals participating in the Swiss Stroke Registry presenting with LS and treated with IVT were included. The study population was then divided into two groups based on patient sex, and a multivariable ordinal logistic regression analysis was performed to uncover sex differences in the modified Rankin Scale (mRS) score at 90 days after stroke.

**Results:**

A total of 413 patients with LS were treated with IVT: 177 (42.9%) women and 236 (57.1%) men. Women were older than men (median age 74 years, 25th–75th percentiles 67–84 years versus 70 years, 25th–75th percentiles 60–80 years, value of p 0.001) and, after adjustment for meaningful variables, showed more frequently increased odds of a higher mRS score at 90 days after stroke (adjusted odds ratio 1.49, 95% confidence interval 1.01–2.19, value of p 0.044).

**Conclusion:**

This study showed that female sex increased the odds of a worse functional response to IVT in patients with LS. Future studies should further elucidate the mechanisms underlying such sex differences.

## 1 Introduction

Lacunar stroke (LS), which accounts for about a quarter of all acute ischaemic strokes (AIS) (1), is a sequela of cerebral small vessel disease (cSVD), a disorder of cerebral microvessels whose underlying pathogenesis remains not completely understood (2–4). Consequently, there are extensive debates on whether LS would benefit from acute reperfusion with intravenous thrombolysis (IVT) (5, 6), which is nowadays the approved treatment for AIS regardless of stroke subtype (7). It was shown that a better functional outcome was present in patients with LS treated with IVT as compared to patients with LS not treated with IVT (8) and that the functional outcome after IVT did not differ in patients with LS compared with other stroke subtypes (9).

LS prevalence is higher with age (10) and seems to be more frequent and severe in men than women (11, 12). Nevertheless, it has not yet been clarified whether there are sex differences in functional outcomes following IVT, specifically in patients with LS. Therefore, this study aimed to assess sex differences in the functional outcome of IVT as a distribution of the modified Rankin Scale (mRS) score at 90 days after stroke among patients with LS from the Swiss Stroke Registry (SSR).

## 2 Methods

### 2.1 Swiss stroke registry

This was a multicenter retrospective cohort study in the SSR, a national registry that collects standardized data from all consecutive patients admitted to certified stroke units and stroke centers across Switzerland. Data collected encompass patient demographics, medical history, medications used before admission, pre-stroke functional status, diagnostic procedures, in-hospital treatments, and outcomes (both during hospitalization and 90 days after stroke). The Clinical Trial Unit of the University Hospital of Basel, Switzerland, manages the database and provides irreversible anonymized data for study purposes. At the time of hospitalization, patients were informed about the collection and utilization of their personal data through informed consent. The registry and the present study were both approved by the responsible ethics committee (CE Req-2020-01042).

### 2.2 Study population

**Figure 1:**
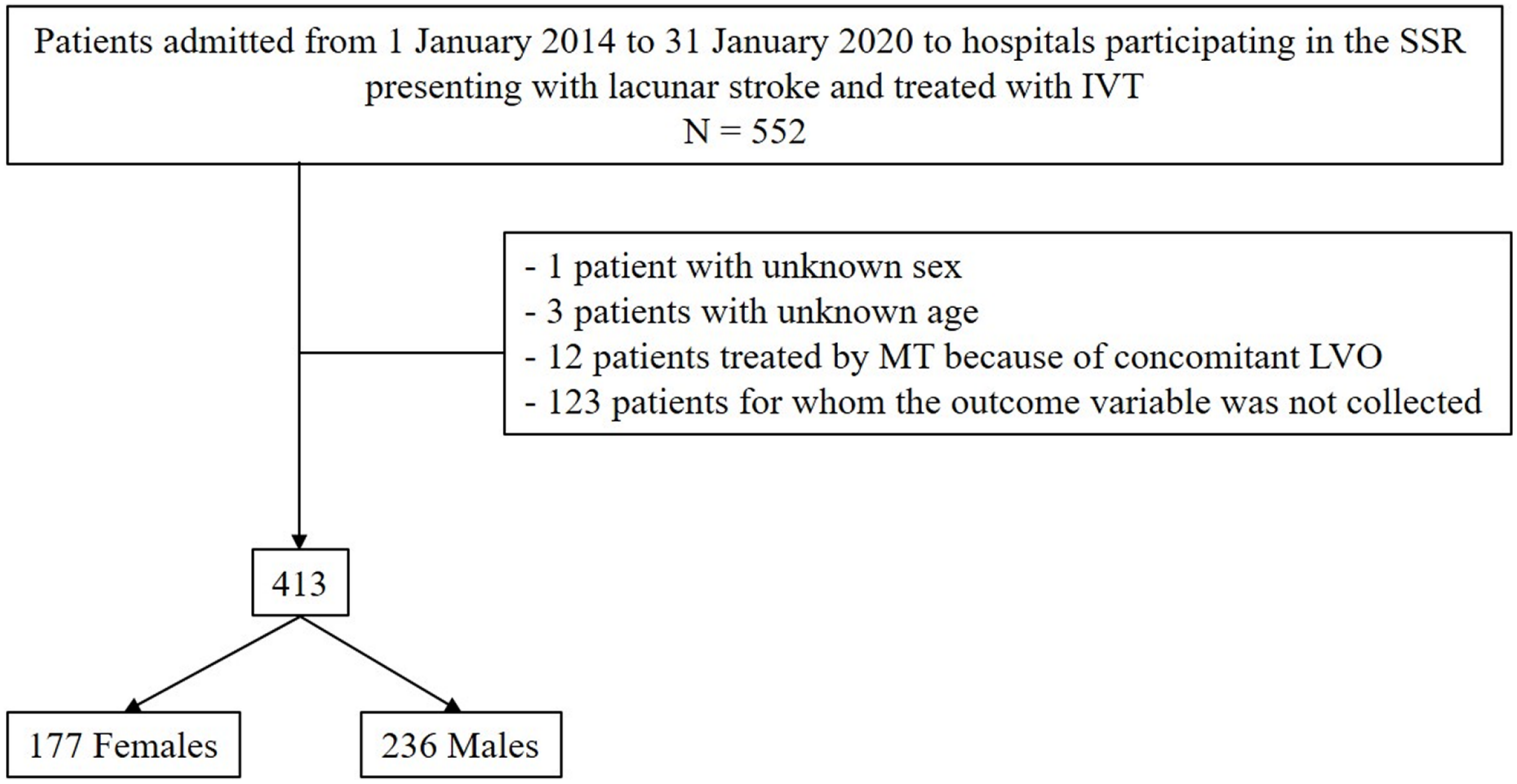
CONSORT diagram showing the selection process of patients included in the study. SSR, Swiss Stroke Registry; IVT, intravenous thrombolysis; MT, mechanical thrombectomy; LVO, large vessel occlusion.

Consecutive patients admitted from 1 January 2014 to 31 January 2020 to hospitals participating in the SSR presenting with LS and treated with IVT were included (Figure 1). In the SSR, the etiology of AIS refers to the TOAST classification (13). The diagnosis of LS was suspected in patients who presented with a recognized lacunar syndrome (e.g., pure motor hemiparesis, pure sensory stroke, ataxic hemiparesis, sensorimotor stroke, and dysarthria-clumsy hand syndrome) or other acute stroke symptoms without cortical involvement and was eventually confirmed upon finding a small subcortical infarct on CT or MRI. The study population was divided into two groups based on patient sex and thereafter compared in terms of patient demographics (age at the time of stroke onset and use of antiplatelets and/or anticoagulants before hospital admission), medical history (previous stroke, previous transient ischemic attack, previous intracranial hemorrhage, hypertension, diabetes, hyperlipidemia, smoking, and atrial fibrillation), pre-stroke functional status (i.e., disability as mRS score ranging from 0 = no symptoms to 6 = death), and stroke severity assessed as National Institutes of Health Stroke Scale (NIHSS) - score at admission (a measure of neurological deficits, ranging from 0 to 42, with higher scores indicating greater stroke severity), in-hospital treatments, and outcomes at 90 days after stroke.

### 2.3 Outcomes

The primary outcome was defined as the mRS score at 90 days after stroke and assessed in an ordinal logistic regression analysis (shift analysis). The safety outcome was in-hospital symptomatic intracerebral hemorrhage (sICH), defined as ICH with ≥4-point worsening of the NIHSS score occurring within 7 days of AIS onset.

### 2.4 Statistical analyses

Quantitative data were presented as absolute numbers with percentages (if categorical) or as median with the 25th and 75th percentiles (if continuous). Comparisons of the different variables according to patient sex were performed with the Mann–Whitney test or chi-squared test, as appropriate. To investigate sex differences in the primary functional outcome of IVT among patients with LS, ordinal logistic regression was used. At first, the association between patient sex and other independent variables of clinical significance and the mRS score at 90 days after stroke was assessed in univariate models. Subsequently, patient sex and variables with a value of *p* ≤0.1 from univariate analyses were included in a multivariable ordinal logistic regression model to provide an overall point estimate as an odds ratio representing a shift in scores on the mRS at 90 days after stroke. All analyses were performed two-sided, and a value of p less than 0.05 was considered statistically significant. Analyses were performed using Statistical Analysis System Software (version 9.4; SA Institute, Cary, NC).

## 3 Results

Out of 5,412 AIS patients treated with IVT, 413 suffered from LS. Supplementary Table S1 shows the distribution of hospitals participating in the SSR from which patients included in the present study were selected. There were 177 women (42.9%) and 236 men (57.1%) (Figure 1). Women were older than men (median age 74 years, 25th–75th percentiles 67–84 years, versus 70 years, 25th–75th percentiles 60–80 years, value of *p* = 0.001). No sex differences were observed in the use of antiplatelets before admission, medical history, pre-stroke disability, or NIHSS score at admission. Female patients showed more frequently worse functional outcomes (both primary and secondary) than male patients, among whom an event of in-hospital sICH occurred (Table 1). No events of sICH occurred among female patients.

**Table 1:** Characteristics of the study population according to patient sex.

Characteristic	Females (*N* = 177)	Males (*N* = 236)	Value of *p*
Age, years, No. (%) <65 65–74 ≥75 median [25th–75th percentiles]	31 (17.5) 58 (32.8) 88 (49.7) 74 [67–84]	79 (33.5) 68 (28.8) 89 (37.7) 70 [60–80]	0.001 <0.0001
Antiplatelets before admission, No. (%) No Yes, single therapy Aspirin Clopidogrel	120 (67.8) 57 (32.2) 49 (27.7) 8 (4.5)	160 (67.8) 76 (32.2) 59 (25.0) 17 (7.2)	1.000 0.539 0.258
Medical history, No.(%) Previous stroke Previous transient ischemic attack Previous intracranial hemorrhage Hypertension Diabetes Hyperlipidemia Smoking Atrial fibrillation	16 (9.0) 12 (6.8) 5 (2.8) 145 (81.9) 35 (19.8) 128 (72.3) 32 (18.1) 4 (2.3)	29 (12.3) 11 (4.7) 2 (0.9) 182 (77.1) 59 (25.0) 185 (78.4) 83 (35.2) 5 (2.1)	0.294 0.353 0.144 0.234 0.210 0.154 0.0001 1.000
Prestroke disability, No. (%) mRS score 0 mRS score 1 mRS score 2 mRS score 3 mRS score 4 mRS score 5	107 (60.5) 26 (14.7) 6 (3.4) 11 (6.2) 1 (0.6) 1 (0.6)	153 (64.8) 23 (9.8) 12 (5.1) 7 (3.0) 4 (1.7) -	0.164
Onset to treatment time (min) median [25th–75th percentiles]	122 (68.9) 207 [131–242]	178 (75.4) 200 [132–228]	0.497
Door to treatment time (min) median [25th–75th percentiles]	166 (93.8) 38 [28–53]	220 (93.2) 39 [29–55]	0.709
NIHSS at admission, No. (%) 0–4 5–10 11–15 16–21 ≥22 median [25th–75th percentiles]	69 (39.0) 95 (53.7) 12 (6.8) 1 (0.6) 0 (0.0) 5 [3.5–7]	108 (45.8) 111 (47.0) 12 (5.1) 4 (1.7) 1 (0.4) 5 [4–7]	0.368 0.496
Functional outcomes at 90 days after stroke, No. (%) Primary mRS score 0 1 2 3 4 5 6 Secondary Functional independence (mRS 0–2)	42 (23.7) 45 (25.4) 33 (18.6) 29 (16.4) 26 (14.7) 0 (0) 2 (1.1) 120 (67.8)	82 (34.8) 61 (25.9) 48 (20.3) 20 (8.5) 18 (7.6) 0 (0) 7 (3.0) 191 (80.9)	0.007 0.002
Safety outcome at 90 days after stroke, No. (%) In-hospital sICH	0 (0)	1 (0.6)	0.430

mRS, modified Rankin Scale; NIHSS, National Institutes of Health Stroke Scale; IVT, intravenous thrombolysis.

Univariate ordinal logistic regression analyses demonstrated that patient sex, use of antiplatelets before admission, previous stroke, medical history of hypertension and hyperlipidemia, as well as pre-stroke disability and NIHSS at admission, were associated with the mRS score at 90 days after stroke (Table 2). After adjustment, female sex was associated with increased odds of presenting a higher mRS score at 90 days after stroke than male sex (adjusted OR 1.49, 95% CI 1.01–2.19, value of p 0.044, Table 2).

**Table 2:** Univariate and multivariable logistic regression analyses.

Independent variables	Distribution of the mRS score at 90 days after stroke (*shift analysis*)			
	Univariate model OR [95% CI]	Value of *p*	Multivariable model Adjusted OR [95% CI]	Value of *p*
Patient sex (female versus male)	0.59 [0.42–0.84]	**0.003**	1.49 [1.01–2.19]	0.044
Patient age (≥65 years versus < 65 years)	0.77 [0.52–1.14]	0.197	–	–
Use of antiplatelets before admission (yes versus no)	0.48 [0.33–0.69]	**<0.0001**	1.47 [0.94–2.29]	0.094
Previous stroke (yes versus no)	0.50 [0.29–0.87]	**0.014**	1.16 [0.59–2.30]	0.663
Previous transient ischemic attack (yes versus no)	1.01 [0.48–2.13]	0.9901	–	–
Hypertension (yes versus no)	0.70 [0.46–1.08]	**0.108**	1.51 [0.91–4.50]	0.112
Diabetes (yes versus no)	0.90 [0.60–1.36]	0.6126	–	–
Hyperlipidemia (yes versus no)	1.39 [0.93–2.08]	**0.105**	0.50 [0.32–0.80]	0.004
Smoking (yes versus no)	1.29 [0.88–1.89]	0.1990	–	–
Prestroke disability (mRS 0 versus mRS 1–6)	0.23 [0.15–0.36]	**<0.0001**	3.62 [2.27–5.76]	<0.0001
NIHSS at admission (<5 versus ≥ 5)	0.55 [0.39–0.78]	**0.001**	1.75 [1.19–2.58]	0.005

OR, odds ratio; CI, confidence interval; mRS, modified Rankin Scale. In bold are variables with a value of *p* ≤ 0.1 that were included in the multivariable regression models.

## 4 Discussion

This study showed that female sex is an independent predictor of a 90-day worse functional outcome in patients with LS treated with IVT. This result, not specifically addressed before, is novel and in line with a previous specific sex-oriented observation performed by our group on IVT efficacy in AIS patients across all stroke subtypes (14).

The reasons behind sex differences in IVT functional outcomes in LS patients are probably multifactorial. First, female patients in our study cohort were older than male patients. Although previous studies have shown that the worse functional outcome after IVT in female patients compared to male patients is not age-dependent (15), additional unmeasured sex- and age-related differences in vascular mechanisms might be involved (such as platelet reactivity or an increase in chronic low-grade systemic inflammation, which has been described to be higher in elderly female patients) (16). Second, the same vascular mechanisms could influence the safety of IVT in LS patients differently between female and male patients. Notably, in our study population, sICH, a feared side effect in patients with small vessel disease, was a rare event. Third, some environmental and social conditions, such as the pre- and post-hospital living situation, might have influenced the worse functional outcome of IVT among female patients with LS. However, despite correcting for the pre-stroke Rankin scale score and choosing a shift analysis as the primary outcome measure, the SSR does not collect detailed information about patient living and social conditions. A fourth factor potentially influencing sex differences in the functional outcome of IVT among LS patients may be related to disparities between male and female patients in acute stroke management, as reported in previous studies (17). However, we found no differences in the timing of treatment between the two sexes. Finally, the different locations of ischemic lesions between female and male patients could have contributed to the worse functional outcome observed in female patients (18). Nevertheless, based on the variables collected within the SSR, we cannot know the specific volumetric measurements of ischemic lesions as well as their location and other neuroimaging details.

Although the national coverage of the SSR may limit the generalizability of the results, the present study evaluates a numerically significant cohort of LS patients with IVT, leveraging the need for future studies aimed at defining the sex-specific mechanisms underlying a worse functional outcome of IVT in female patients with LS.

## Data availability statement

The raw data supporting the conclusions of this article will be made available by the authors, on request.

## Ethics statement

The study was approved by Comitato Etico Canton Ticino (CE Req-2020-01042). The studies were conducted in accordance with the local legislation and institutional requirements. Written informed consent for participation was not required from the participants or the participants’ legal guardians/next of kin in accordance with the national legislation and institutional requirements.

## Author contributions

PW: Data curation, Validation, Visualization, Writing – original draft. RN: Data curation, Formal analysis, Investigation, Software, Validation, Visualization, Writing – original draft. AP: Data curation, Formal analysis, Investigation, Methodology, Software, Writing – review & editing. GB: Resources, Writing – review & editing. DS: Resources, Writing – review & editing. PM: Resources, Writing – review & editing. KN: Resources, Writing – review & editing. LB: Resources, Writing – review & editing. GK: Resources, Writing – review & editing. JN: Resources, Writing – review & editing. TN: Resources, Writing – review & editing. AL: Resources, Writing – review & editing. SW: Resources, Writing – review & editing. LS: Resources, Writing – review & editing. FM: Resources, Writing – review & editing. BR: Resources, Writing – review & editing. NP: Resources, Writing – review & editing. SR: Resources, Writing – review & editing. MM: Resources, Writing – review & editing. EC: Resources, Writing – review & editing. UF: Resources, Writing – review & editing. GM: Resources, Writing – review & editing. CC: Conceptualization, Funding acquisition, Investigation, Project administration, Resources, Supervision, Validation, Visualization, Writing – review & editing.
