# Supplementary material for: Sex differences in functional outcomes of intravenous thrombolysis among patients with lacunar stroke
Source: Front Neurol. 2024 Feb 13;15:1341423. doi: 10.3389/fneur.2024.1341423 (PMC10914024; doi:10.3389/fneur.2024.1341423)
Supplement: Supplementary file 1 [file Table_1.docx]

**Supplementary Table 1**. Distribution of hospitals participating in the Swiss Stroke Registry from which patients included in the present study were selected.

| **Hospital** | **N (%)** |
| --- | --- |
| Universitätsspital Basel | 90 (21.8) |
| Kantonsspital St. Gallen | 50 (12.1) |
| Bern Inselspital | 39 (9.4 |
| Kantonsspital Münsterlingen | 30 (7.3) |
| Kantonsspital Aarau | 28 (6.8) |
| Kantonsspital Winterthur | 25 (6.1) |
| CHUV Lausanne | 23 (5.6) |
| Kantonsspital Luzern | 23 (5.6) |
| HUG Genève | 18 (4.4) |
| Universitasspital Zurich | 18 (4.4) |
| Ospedale Regionale di Lugano | 13 (3.2) |
| Kantonsspital Graubünden | 12 (2.9) |
| Bürgerspital Solothurn | 11 (2.7) |
| Spital Sarganserland Grabs | 10 (2.4) |
| Kninik Hirslanden Zurich | 8 (1.9) |
| Hopital Fribourg | 5 (1.2) |
| Spitalzentrum Biel | 5 (1.2) |
| Stadspital Triemli Zurich | 3 (0.7) |
| Hopital Nyon | 1 (0.2) |
| Spital Limmattal | 1 (0.2) |
